# Application of mNGS in the study of pulmonary microbiome in pneumoconiosis complicated with pulmonary infection patients and exploration of potential biomarkers

**DOI:** 10.3389/fcimb.2023.1200157

**Published:** 2023-07-21

**Authors:** Xingya Yuan, Linshen Xie, Zhenzhen Shi, Min Zhou

**Affiliations:** ^1^ Department of Respiratory Medicine, West China Fourth Hospital, Sichuan University, Chengdu, Sichuan, China; ^2^ Dinfectome Inc., Nanjing, Jiangsu, China

**Keywords:** pneumoconiosis, microbiome, metagenomic next-generation sequencing, pulmonary infection, biomarker

## Abstract

**Background:**

Pneumoconiosis patients have a high prevalence of pulmonary infections, which can complicate diagnosis and treatment. And there is no comprehensive study of the microbiome of patients with pneumoconiosis. The application of metagenomic next-generation sequencing (mNGS) fills the gap to some extent by analyzing the lung microbiota of pneumoconiosis population while achieving accurate diagnosis.

**Methods:**

We retrospectively analyzed 44 patients with suspected pneumoconiosis complicated with pulmonary infection between Jan 2020 and Nov 2022. Bronchoalveolar lavage fluid (BALF) specimens from 44 patients were collected and tested using the mNGS technology.

**Results:**

Among the lung microbiome of pneumoconiosis patients with complicated pulmonary infection (P group), the most frequently detected bacteria and fungi at the genus level were *Streptococcus* and *Aspergillus*, at the species level were *Streptococcus pneumoniae* and *Aspergillus flavus*, respectively, and the most frequently detected DNA virus was *Human gammaherpesvirus 4*. There was no significant difference in α diversity between the P group and the non-pneumoconiosis patients complicated with pulmonary infection group (Non-P group) in pulmonary flora, while *P<* 0.01 for β diversity analysis, and the differential species between the two groups were *Mycobacterium colombiense* and *Fusobacterium nucleatum*. In addition, we monitored a high distribution of *Malassezia* and *Pneumocystis* in the P group, while herpes virus was detected in the majority of samples.

**Conclusions:**

Overall, we not only revealed a comprehensive lung microbiome profile of pneumoconiosis patients, but also compared the differences between their microbiome and that of non-pneumoconiosis complicated with pulmonary infection patients. This provides a good basis for a better understanding of the relationship between pneumoconiosis and microorganisms, and for the search of potential biomarkers.

## 1 Introduction

Pneumoconiosis is a group of lung diseases caused by the inhalation of inorganic mineral particles, usually because of certain occupations. Its main pathological features include chronic lung inflammation and progressive pulmonary fibrosis (Perret et al., 2017), which can lead to respiratory and/or cardiac failure and eventually death. Pneumoconiosis is prevalent worldwide, with more than 60,000 new cases reported worldwide in 2017 (Shi et al., 2020). With the development and optimization of the industry in recent years, the pneumoconiosis population has decreased from 23.33% before 1970 to 2.29% in 2020 (Liu et al., 2022). However, the mortality rate of pneumoconiosis is relatively high (GBD 2017 Disease and Injury Incidence and Prevalence Collaborators, 2018; GBD 2013 Mortality and Causes of Death Collaborators, 2015), which is a serious threat to global public health.

Patients with pneumoconiosis are susceptible to microbial invasion such as *Mycobacterium tuberculosis* (Jun et al., 2013), *nontuberculous mycobacteria* (*NTM*) (McGrath and Bardsley, 2009) and *Aspergillus*(Vangara et al., 2022), leading to pulmonary infection. And many patients with advanced pneumoconiosis die of respiratory failure due to pulmonary infections (Barnes et al., 2019; Qi et al., 2021). Traditional etiologic methods such as microscopy, smear, and culture have low sensitivity, subjectivity, and contamination, which can lead to missed or false detection and affect patient outcomes (Dahyot et al., 2017). It is very important for patients with pulmonary infections to identify the etiology and use accurate drugs, especially for patients with lung damage such as pneumoconiosis. Many studies have revealed that the abundance and composition of microbial communities vary in different body habitats, with strong links to health status and human disease (Dickson et al., 2020; Wu et al., 2020). However, current analysis of bacterial community diversity in pneumoconiosis mostly uses sputum culture and 16S rRNA, which are not sufficient for microbiome analysis, and in most cases, microorganisms cannot be identified to species level (Mingjing Chen et al., 2017; Zhimin Ma, 2020; Druzhinin et al., 2022).

Metagenomic next-generation sequencing (mNGS) has the advantages of broad coverage, unbiased and unpredictable, and can simultaneously identify bacteria, fungi and viruses in a single sample (Chiu and Miller, 2019; Chen et al., 2021; D’Humières et al., 2021). It has been widely used in clinical practice in recent years, playing an important role in assisting clinical diagnosis, guiding rational drug use, reducing patient burden, and improving patient clinical outcome (Qian et al., 2020). In addition, mNGS does not require culture and pathogen detection results are typically available within 24-48 hours and are less susceptible to antibiotics than culture (Miao et al., 2018). Early diagnosis of pneumoconiosis complicated with pulmonary infection patients is very important due to the poor prognosis (Barnes et al., 2019; Qi et al., 2021), while the use of mNGS technique has not been reported for these patients. This study retrospectively examines pulmonary microbiome (bacterial, fungal, viral) characteristics in pneumoconiosis patients with pulmonary infection (P group), compares the pulmonary microbiome to non-pneumoconiosis patients with pulmonary infection (Non-P group), analyzes differential microbiome, and explores potential diagnostic biomarkers of pneumoconiosis.

## 2 Methods

### 2.1 Study population

**Figure 1:**
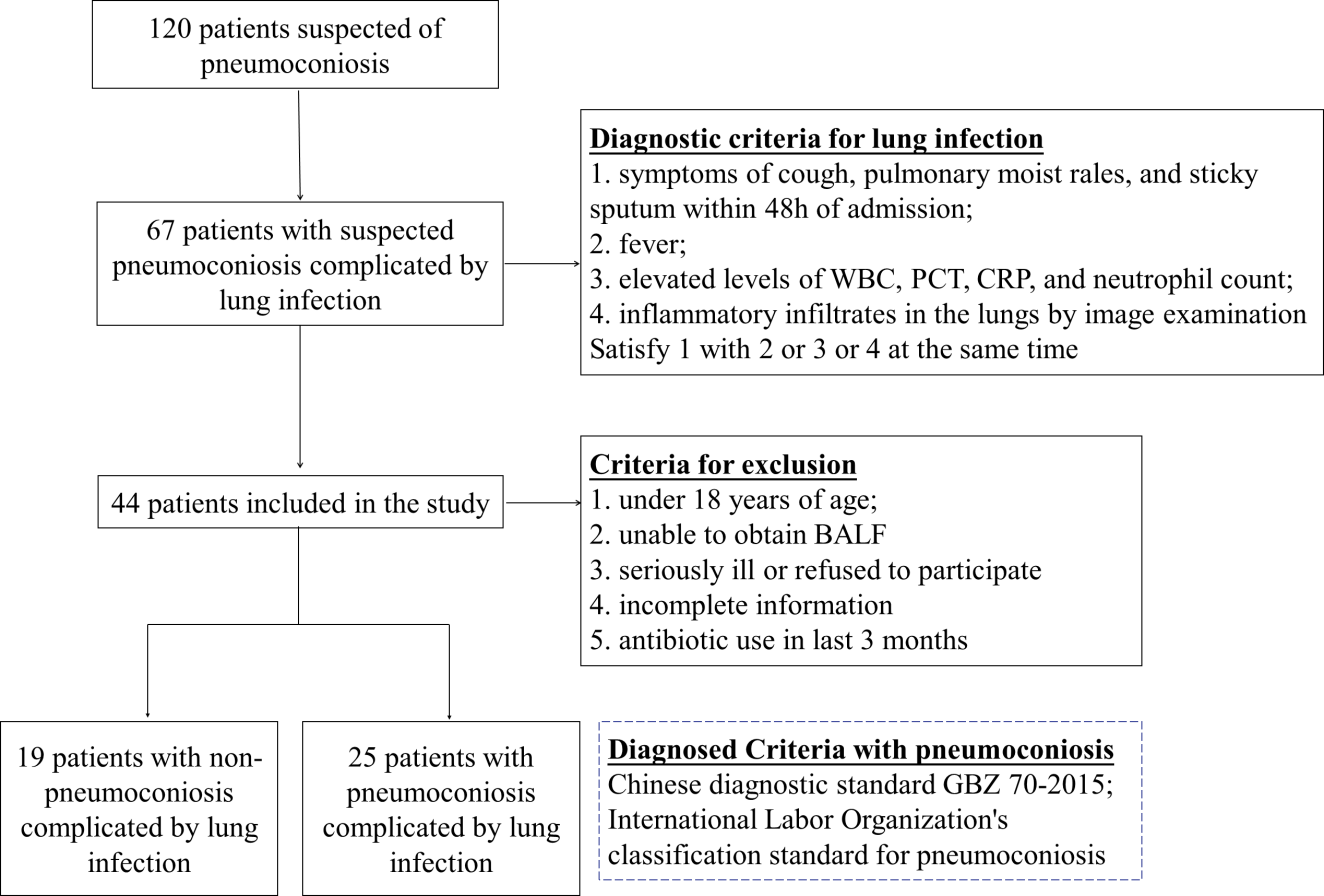
Inclusion and exclusion flowchart of study.

Patients with suspected pneumoconiosis complicated with pulmonary infection were recruited, the diagnostic criteria for pulmonary infection was shown in **Figure 1** (Cao et al., 2018; Shi et al., 2019), and pneumoconiosis was diagnosed with pneumoconiosis by the Chinese diagnostic standard GBZ 70-2015 and the International Labor Organization’s classification standard for pneumoconiosis (Honma et al., 2004), Recruitment was carried out at a single site in West China Fourth Hospital Sichuan University, Chengdu between Jan 2020-Nov 2022, Patients who were under 18 years of age, unable to obtain bronchoalveolar lavage fluid (BALF), and had incomplete information were excluded from our study. Besides, some of the collected samples have been tested by G test, GM test or culture before mNGS. Data were collected on the demographics, underlying diseases and clinical features of the patients enrolled and were listed in **Table 1**.

**Table 1:** Patient and sample characteristics including biochemical parameters, underlying disease and clinical features.

	Pneumoconiosis (P) (n=25)	Non-Pneumoconiosis (Non-P) (n=19)
Age(years) (mean ± SD)	51.68 ± 11.51	62.2 ± 14.4
Gender		
Male	25	14
Female	0	5
Inflammatory index		
WBC(×10^9^/L) (mean ± SD)	8.32 ± 2.41	8.85 ± 5.35
PCT(μg/L) (mean ± SD)	0.21 ± 0.09	0.19 ± 0.07
CRP (mg/L) (mean ± SD)	57.73 ± 78.00	71.86 ± 70.46
Neutrophils(×10^9^/L) (mean ± SD)	6.51 ± 2.49	6.94 ± 5.34
Lymphatic cells(×10^9^/L) (mean ± SD)	1.05 ± 0.59	1.18 ± 0.44
Working years (years) (mean ± SD)	10.76 ± 9.78	/
Underlying disease		
Tuberculosis/history of tuberculosis (n)	6	1
Hypertension (n)	5	2
Hepatitis B (n)	3	1
chronic cor pulmonale	3	0
type 2 diabetes (n)	2	2
chronic obstructive pulmonary disease (n)	2	1
Cancer (n)	0	2
Clinical characterization		
Fever (n)	6	7
Cough (n)	24	18
Expectoration (n)	23	14
Dyspnea (n)	5	1
Hemoptysis (n)	8	4

SD, standard deviation; Working years, Patient’s years of pneumoconiosis-related work; WBC, White Blood Count; PCT, Procalcitonin; CRP, C-reactive protein; Neutrophils, Neutrophil count, Lymphatic cells, Lymphocyte count.

### 2.2 Specimen collection

BALF was obtained from 44 participants. The purpose of collecting BALF is to make an etiologic diagnosis of the patient’s infection. Samples were collected from patients according to standard procedures (Levy et al., 2018). After local anesthesia of the patient’s throat, the fiberoptic bronchoscope was introduced. The lung was lavaged with room temperature sterile saline several times through the fiberoptic bronchoscope, 20-60 mL each time. 10 mL of the sample was removed from the recovered solution, place 2 mL of it into a sampling tube with RNA protection solution (Sigma-Aldrich) and the rest into a sterile nucleic acid-free DNA sampling tube and store immediately at -80°C.

### 2.3 Sample DNA and RNA extraction

BALF DNA was extracted using methods previously described (Mac Aogáin et al., 2021; Ju et al., 2022), take 50 μL of proteinase k and 1 mL of BALF sample, digest at 60°C for 20 min, and then leave at 4°C for 5 min to lower the reaction temperature. Transfer the sample to a sterile test tube and centrifuge briefly followed by DNA extraction using the TIANamp Magnetic DNA Kit (DP710-t2, Tiangen, China) according to the manufacturer’s protocol. Sputum was liquefied by 0.1% DTT (dithiothreitol) for 20 min at 56°C before extraction. The QIAamp Viral RNA Mini Kit (Qiagen) was used to extract RNA from the BALF (Langelier et al., 2018).

DNA libraries were prepared using the KAPA Hyper Prep Kit (KAPA Biosystems) according to the manufacturer’s protocol. Libraries were constructed after Qubit quantification. For RNA extraction samples, rRNA was removed from total RNA and libraries were constructed after purification as described for DNA library construction. Agilent 2100 was used for quality control and then DNA libraries were sequenced on the Dif seq platform for 50 bp paired end sequencing (Dinfectome Medical Technology Inc, Nanjing, China).

### 2.4 Bioinformatics analysis

For pathogen identification, we used an in-house developed bioinformatics pipeline (Zeng et al., 2022). Briefly, low quality reads, adapter contamination, duplicated and shot (length <36 bp) reads were removed to generate high quality sequencing data. Sequences from the human host were identified by mapping to the human reference genome (hs37d5) using the bowtie2 software (Langmead and Salzberg, 2012). Reads that could not be mapped to the human genome were retained. They were aligned to the microorganism genome database for pathogen identification. Our microorganism genome database contained the genome sequences of bacteria, fungi, viruses, and parasites (can be downloaded from https://www.ncbi.nlm.nih.gov/) (Wood et al., 2019).

### 2.5 Interpretation and reporting

The mNGS pathogen detection pipeline was described in previous studies (Miao et al., 2018; Miller et al., 2019; Qian et al., 2020; Zeng et al., 2022; Chen et al., 2023; Xu et al., 2023), and the criteria for detection positivity were as follows: 1) at least one species-specific read for *Mycobacterium tuberculosis*, *Nocardia* and *Legionella pneumophila* detection; 2) for other bacteria, fungi, virus, and parasites, at least three unique reads were needed; 3) pathogens were excluded if the ratio of microorganism reads per million of a given sample versus NTC was < 10.

### 2.6 Statistics analysis

The statistical analysis was carried out using the R software (v4.2.1) (R Core Team, 2021). Alpha diversity was estimated by Shannon index and Simpson index based on the taxonomic profile of each sample. Beta diversity was assessed by Bray-Curtis measure. PERMANOVA was performed using the R package “vegan” to analyze the Bray-Curtis distance in different P and Non-P groups. In all cases, two-tailed analysis was performed and considered. Differences were regarded as significant at *P* < 0.05. Differential relative abundance of taxonomic groups at the genus/species level between groups was tested using the Kruskal-Wallis rank sum test (R package “kruskal.test”) (Kruskal and Wallis, 1952). Statistical analyses and plots were processed by using SPSS statistical software (IBM SPSS Statistics for Windows, Version 25.0. Armonk, NY, United States) and GraphPad Prism software (GraphPad Prism version 8.0.2 for Windows, GraphPad Software, San Diego, CA, United States).

## 3 Results

### 3.1 General information of study participants

120 patients suspected of pulmonary infection and pneumoconiosis were screened, 44 eligible patients were included in the final analysis. Including 25 patients with pneumoconiosis and 19 patients with non-pneumoconiosis, 25 patients with pneumoconiosis and 19 patients with non-pneumoconiosis underwent bronchoscopy to obtain BALF. In terms of patient composition, all participants in the study were male and no female patients were enrolled in pneumoconiosis due to occupational characteristics. The main types of dusts causing pneumoconiosis according to clinical data were production dust (indoor work), mineral dust (coal mine, drilling related work), and the average number of years patients were exposed to such work was 10.76 years.

### 3.2 Characteristics of the pulmonary microbiome of pneumoconiosis patients

**Figure 2:**
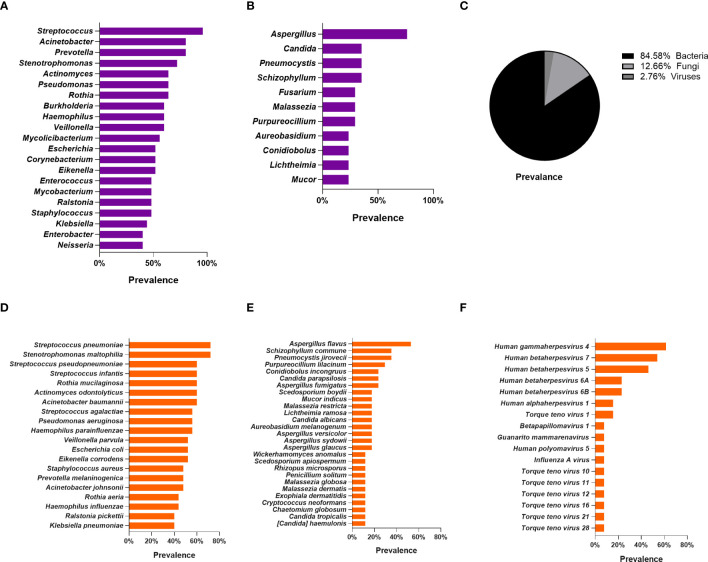
Lung microbiome of patients with pneumoconiosis complicated with pulmonary infection (BALF). **(A)** Distribution of bacteria at the genus level. **(B)** Distribution of fungi at the genus level. **(C)** Distribution pie chart of detected bacteria, fungi, and viruses at the species level. **(D)** Distribution of bacteria at the species level. **(E)** Distribution of fungi at the species level. **(F)** Distribution of Viruses at the species level.

We plotted bar charts based on the frequency of species detection in pneumoconiosis patients, with the top 10 genera and top 20 species detected. In BALF samples, 521 bacterial species, 78 fungi species, and 17 viral species were detected in the pneumoconiosis patient group. At the genus level, the top three bacteria detected were *Streptococcus* (96%), *Acinetobacter* (80%), and *Prevotella* (80%). *Aspergillus* (76.47%), *Candida* (35.29%), *Pneumocystis* (35.29%) for fungi. At the species level, the top 3 bacterial species detected were *Streptococcus pneumoniae* (72%, relative abundance 0.040%), *Stenotrophomonas maltophilia* (72%, relative abundance 0.036%) and *Rothia mucilaginosa* (60%, relative abundance 0.047%), based on frequency of detection and relative abundance of species detected. In terms of fungal detections, the top 3 were *Aspergillus flavus* (52. 94%), *Pneumocystis jirovecii* (35.29%), and *Schizophyllum commune* (35.29%). In addition, we revealed that herpes viruses were detected more frequently in pneumoconiosis patients, with *Human gamma herpesvirus type 4* detected in 61.54% of all patients, and *Human betaherpesvirus type 7* and *Human beta herpesvirus type 5* detection rates of 53.85% and 46.15%, respectively. Meanwhile, RNA viruses were found in two patients, *Human coronavirus NL63*, *Human respiratory virus 3* and *Rhinovirus A*, respectively. Specific detections can be found in **Figure 2**. Also, we counted the results of conventional microbiological testing of BALF samples. 22 BALF samples were cultured, G test and GM test simultaneously, and 15 samples were cultured only, however, all of these results were negative based on clinical judgment.

### 3.3 Microbiota analysis between P and Non-P groups

Analysis of microbiome differences in pneumoconiosis patients and non-pneumoconiosis patients will help understand the relationship between microbes and pneumoconiosis and identify biomarkers relevant to pneumoconiosis diagnosis.

**Figure 3:**
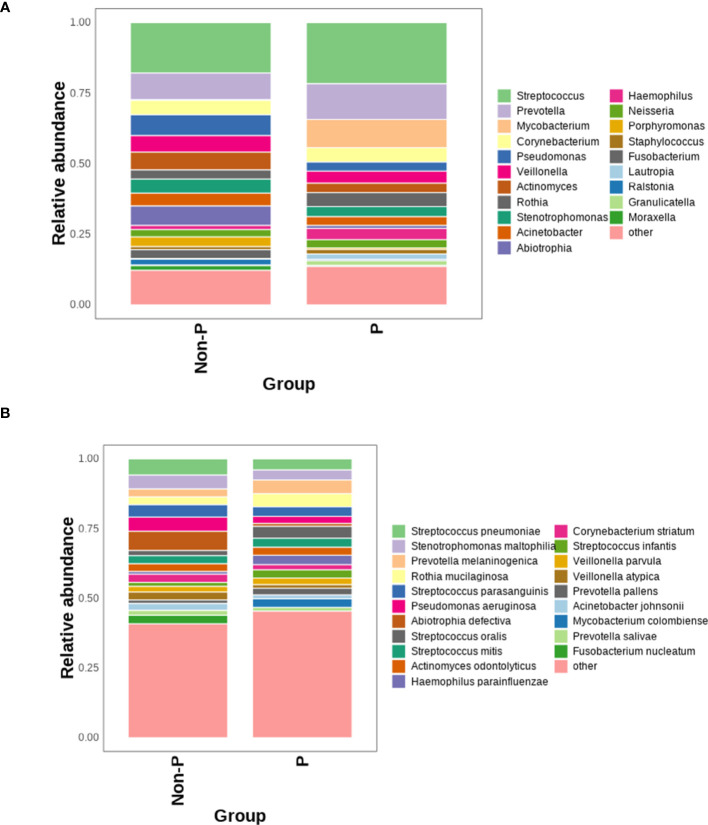
Comparison of the relative abundance of microorganisms between P and Non-P groups. **(A)** Distribution of bacteria at the genus level in the P and Non-P groups. **(B)** Distribution of bacteria at the species level in the P and Non-P groups.

Bar graphs were plotted based on the relative abundance of detected species, as shown in **Figure 3**, and the species with the highest relative abundance at the genus level in the P and Non-P groups were detected as *Streptococcus*. Among the top 10 genera in terms of relative abundance, the relative abundance of *Streptococcus*, *Prevotella*, *Mycobacterium* and *Rothia* in the P group was higher than that Non-P group, while all other genera had higher relative abundance in the Non-P group, the relative abundance of *Corynebacterium* was essentially equal between the two groups.

At the species level, among the top 10 species by relative abundance, *Prevotella melaninogenica*, *Rothia mucilaginosa*, *Streptococcus oralis*, *Streptococcus mitis* were detected in higher relative abundance in the P group than Non-P group, while the remaining species had higher relative abundance in the Non-P group. Among them, *Pseudomonas aeruginosa* was usually associated with poor patient prognosis (Wang et al., 2019), while *Abiotrophia defectiva* was normal in the oral, genitourinary, and intestinal tracts, may cause sometimes serious infections in humans (Li et al., 2022).

**Figure 4:**
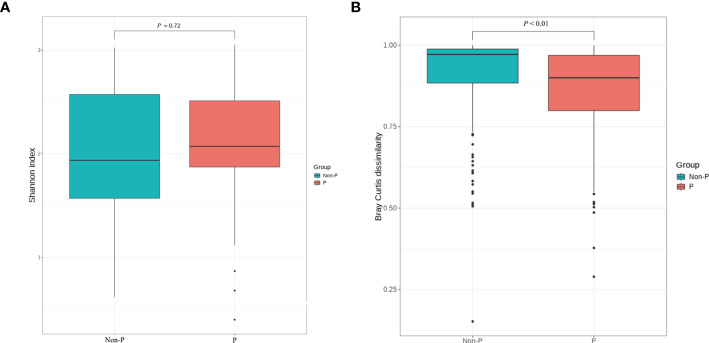
α and β diversity analysis between P and Non-P groups. **(A)** Shannon Index analysis. **(B)** Bray Curtis dissimilarity analysis.

To analyze the differences in species diversity between the groups, α-diversity and β-diversity were used. The findings proved that there was no significant difference in ACE, Chao1, Shannon or Simpson between the two groups (*P* > 0.05, only the Shannon Diversity Index results were shown), indicating similar species variety. The difference in species between groups was analyzed with β diversity, and *P* < 0.01, suggesting that there was a remarkable difference in species between groups and the grouping was meaningful, as shown in **Figure 4**.

**Figure 5:**
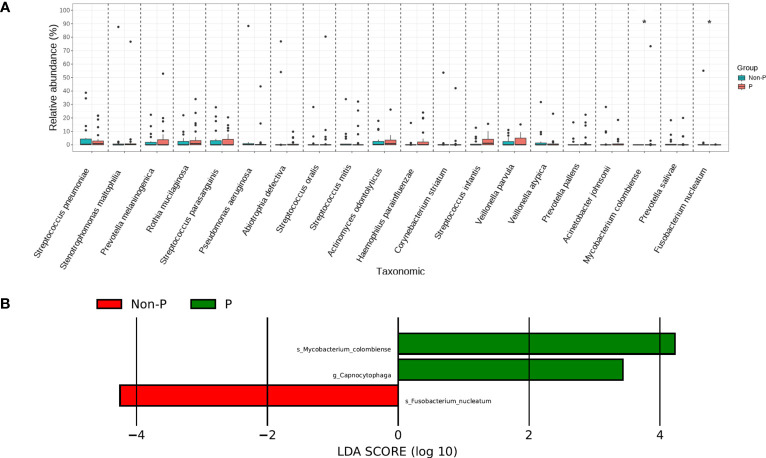
Species analysis of differences between P and Non-P groups. **(A)** Analysis of significant differences species. **(B)** LEfSe analysis.

We tested species differences between P and Non-P groups at phylum, genus and species level. No conspicuous differences were found in the phylum and genus between the groups, However, the distribution of species differed dramatically. *Mycobacterium colombiense* (*M. colombiense*) and *Fusobacterium nucleatum* (*F. nucleatum*) were evidently different in their presence (**Figure 5A**), with the former being detected mainly in pneumoconiosis patients and the latter mainly in non-pneumoconiosis patients. The study also used LEfSe analysis to explore species that differed strikingly between groups (**Figure 5B**), with only three species differing between the two groups, including one at the genus level and two at the species level (i.e. the two different species mentioned above), the genus *Capnocytophaga* was enriched in the P group.

**Figure 6:**
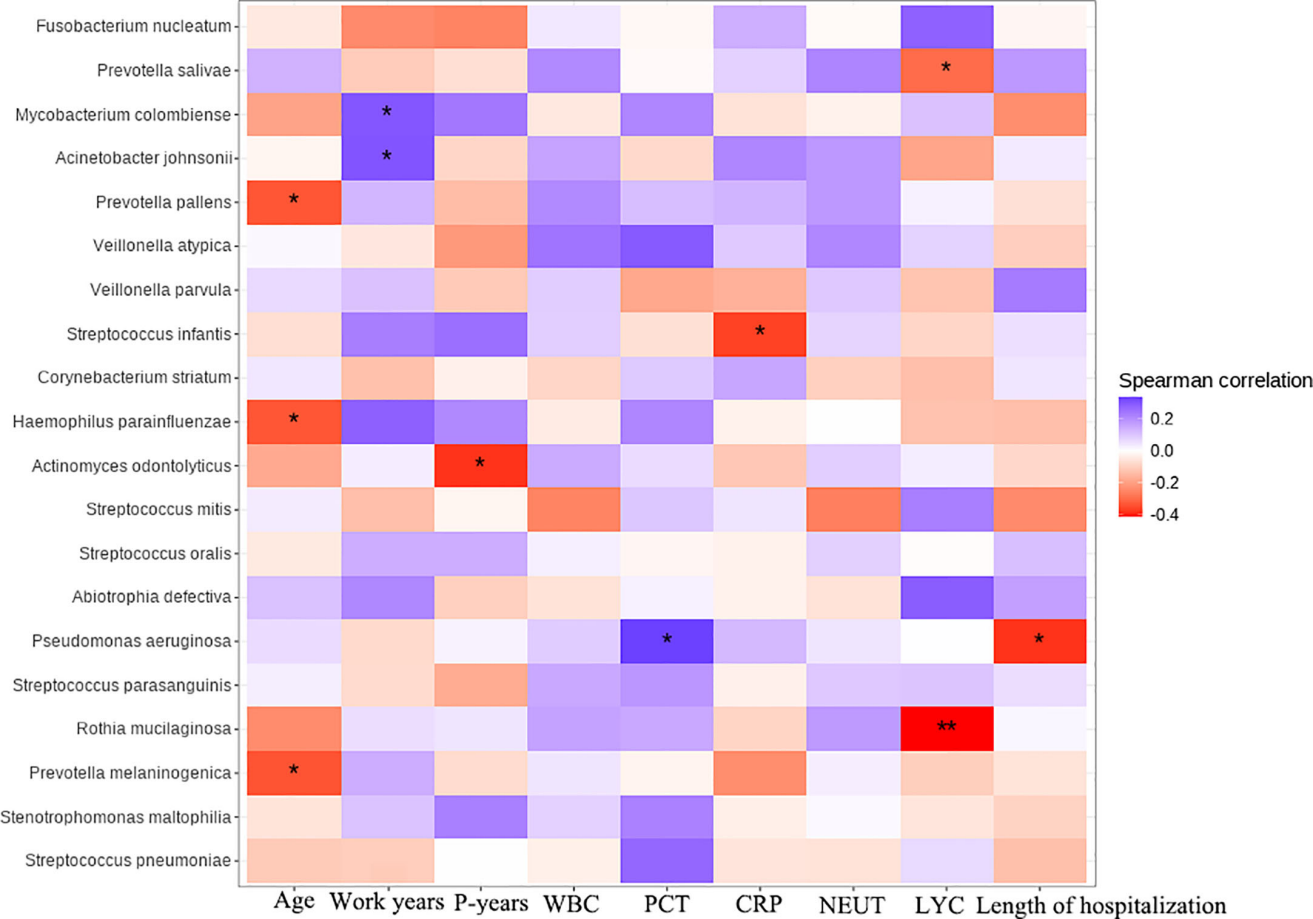
Clinical and microbial correlation analysis, Work years, Patient’s years of pneumoconiosis-related work; P-years, Pneumoconiosis years; WBC, White Blood Count; PCT, Procalcitonin; CRP, C-reactive protein; NEUT, Neutrophil count; LYC, Lymphocyte count. The symbol * represent significance p < 0.05.

Sperman correlation analysis was performed to explore the correlation between clinical parameters such as patient’s age, pneumoconiosis years, and inflammatory indicators at admission with significantly different species and the top 18 species in terms of relative abundance (for a total of 20 species, **Figure 6**). *Prevotella*, *Actinomyces* and *Rothia* were common colonizing organisms in the mouth, *Prevotella melaninogenica*, *Prevotella pallens*, *Actinomyces odontolyticus*, *Rothia mucilaginosa* and other oral bacteria were distinctly and negatively correlated with patients’ age, pneumoconiosis years and lymphocyte count, which may mean that the abundance of these microorganisms decreases as pneumoconiosis progresses. *M. colombiense* was positively correlated with years of work related to pneumoconiosis, suggesting that the likelihood of *M. colombiense* infection increased with the progression of pneumoconiosis, while we observed that the relative abundance of *Pseudomonas aeruginosa* was positively correlated with the length of hospitalization of pneumoconiosis patients, which seemed somewhat unusual and might be related to the small number of patients enrolled.

### 3.4 Comparison of fungi and virus detection in P and Non-P

**Figure 7:**
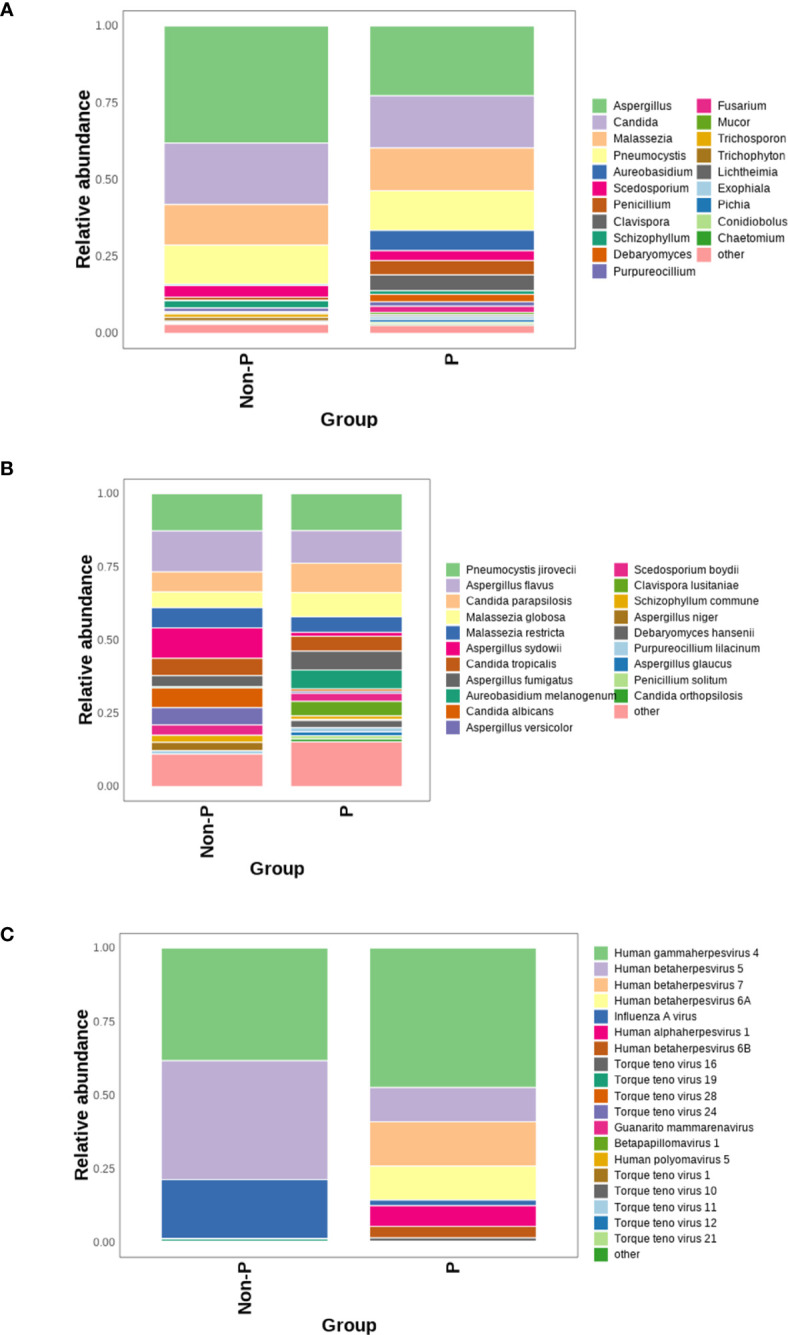
Analysis of viruses and fungi in P and Non-P groups. **(A)** Distribution of fungi at the genus level in the P and Non-P groups. **(B)** Distribution of fungi at the species level in the P and Non-P groups. **(C)**. Distribution of viruses at the genus level in the P and Non-P groups.

The mNGS technology can identify and detect bacteria, fungi and viruses in the same sample, which is more conducive to a fully revealed microbiome signature. The top 20 genera/species were plotted in terms of relative abundance of species detected in the P group, as shown in the **Figure 7**. At the genus level, the top four genera detected were *Aspergillus*, *Candida*, *Malassezia* and *Pneumocystis*. Among them, more *Malassezia* and *Pneumocystis* were distributed in the P group, while *Aspergillus* and *Candida* were more dominant in the Non-P group. At the species level, among the top five detected species, *Aspergillus sydowii*, *Aspergillus versicolor*, *Candida albicans* were higher in the Non-P group than in the P group, while *Aureobasidium melanogenum*, *Clavispora lusitaniae* were higher in the P group. The viruses detected were displayed in **Figure 7C** below, with more viruses detected in the P group, while *Human gammaherpesvirus 4*, *Human betaherpesvirus 5*, *Influenza A virus* were mainly detected in the Non-P group. *Human gammaherpesvirus 4*, *Human betaherpesvirus 5*, *Human betaherpesvirus 7* and *Human betaherpesvirus 6A* were mainly detected in the P group. The *Human gammaherpesvirus* or *Human betaherpesvirus* mentioned above belong to the same family, *Herpesviridae*.

## 4 Discussion

In this study, mNGS technology was used to comprehensively reveal the pulmonary microbiome of pneumoconiosis patients, including the characteristics of bacteria, fungi and viruses, through BALF samples, and compare the differences in the lung microbiome between the P and Non-P groups so as to compare the microbial differences between the two groups for the exploration of potential biomarkers. To our knowledge, this current study is the first to investigate the lung microbiome of pneumoconiosis patients using a comprehensive and systematic mNGS technique and is also the first study to reveal differences in the lung microbiome of patients with pneumoconiosis versus non-pneumoconiosis.

Due to the chronic progressive disease of pneumoconiosis and the usual damage to the respiratory mucosa in pneumoconiosis patients, pneumoconiosis patients have a high probability of the lower respiratory tract (Xin and Zhang, 2017). Our study is the first to use mNGS to reveal the lung flora of pneumoconiosis complicated with pulmonary infection patients. In a previous study, Druzhinin et al. employed 16S to analyze the microbial composition of sputum samples from coal workers’ pneumoconiosis (CWP) and observed a significant increase in the abundance of *Streptococcus* compared to the healthy group (Druzhinin et al., 2022). In addition, Li et al. analyzed the intestinal flora of pneumoconiosis patients and demonstrated a remarkable increase of *Prevotella* abundance in the pneumoconiosis group compared to the control group (Li et al., 2022). Similarly, we monitored higher abundance of *Streptococcus* and *Prevotella* in BALF samples from the P group compared to the Non-P group, however, the differences between both groups were non-significant, which we analyzed may be related to differences in sample type, as well as the fact that sputum specimens are susceptible to oral colonization flora compared to BALF samples.

Infections caused by fungi are gradually increasing in the clinic due to the irrational use of antibiotics and the increased use of hormonal drugs. Aspergillus is one of the main pathogens causing invasive fungal diseases, as well as chronic pulmonary aspergillosis, may worsen symptoms in advanced chronic obstructive pulmonary disease (COPD) (Hammond et al., 2020), and is associated with high mortality (Vandewoude et al., 2004). *Aspergillus fumigatus* is the most common agent of invasive aspergillosis and has been widely studied and reviewed (Dewi et al., 2021; Deng et al., 2023). However, *Aspergillus flavus* is the most frequently detected fungi in our studies of the pulmonary microbiome of pneumoconiosis patients, it can produce the most carcinogenic mycotoxin aflatoxins and cause aspergillosis in immune-compromised patients. Meanwhile, *in vivo* experimental studies have shown that the fungi is more toxic than *Aspergillus fumigatus* and other *Aspergillus* species in terms of time to death and initial inoculum in normal and immunocompromised experimental mice (Rudramurthy et al., 2019). The G test is widely used for invasive fungal infections (Lu et al., 2011; Li et al., 2015), while the GM test can further identify invasive aspergillosis for early diagnosis (Guo et al., 2010). In our study, some of the BALF samples were subjected to both G test and GM test, however, their negative results indicated the limitations of the traditional testing method to some extent, while the culture of BALF samples seemed to be unsatisfactory. Due to the specificity of the pneumoconiosis patient population, most of the patients have been on long-term antibiotic and antifungal medication prior to the relevant tests, which we speculate may be one of the reasons for the unsatisfactory results of the traditional tests, while some studies have reported that the detection rate of mNGS is relatively less affected by the use of antibiotics compared to the traditional testing modalities (Miao et al., 2018; Diao et al., 2021). Beyond this, a combination of guidelines and consensus, mNGS will be conducted when conventional tests fail to clarify the pathogen, which may be due to the high cost limitations of sequencing (Chinese Thoracic Society, 2023). We expect the reduced cost of mNGS technology in the future to make this tool more accessible, especially for low resource settings where the burden of infectious diseases is high and the availability of many pathogen-specific assays is low (Ramachandran et al., 2022).

The high detection rate of *Mycobacterium* in pneumoconiosis patients has been confirmed in large number of studies, including *Mycobacterium tuberculosis* and *NTM* (Kim et al., 2009). *M. colombiense* is mainly found in patients with pneumoconiosis and is an emerging species in the complex group of *Mycobacterium avium*, characterized by acid resistance, immobility, rod-shaped structure, and slow growth. It was first isolated and described by Murcia in 2006, and can be isolated in blood, sputum, and lymph nodes (Murcia et al., 2006; Tang et al., 2023). The bacterium is prone to cause severe pulmonary infection in immunodeficient or immunosuppressed patient (Yu and Jiang, 2021), disseminated diseases (Pena et al., 2019), ganglionar mycobacteriosis related diseases (Larry et al., 2019), and disseminated diseases associated with immunocompetent patients have also been reported (Esparcia et al., 2008; Tang et al., 2023). Cases of the bacterium have been reported in Europe, America, and Asia (Vuorenmaa et al., 2009; Poulin et al., 2013; Gao et al., 2014). However, there is a lack of attention to this bacterium, and it is often ignored in clinical diagnosis (Van Ingen et al., 2018). Our study identified for the first time that *M. colombiense* was substantially enriched in BALF samples of P group, which may be related to lung damage of these patients. The detection of this bacterium requires special attention as it could be a potential biomarker to distinguish pneumoconiosis from non-pneumoconiosis. However, this result has not been reported in previous studies of flora associated with pneumoconiosis (Druzhinin et al., 2022; Li et al., 2022), which may be due to differences in sample types. Although our study inaugurally evaluates the lung microbiota of pneumoconiosis complicated with pulmonary infection patients and reveals a notable enrichment of *M. colombiense* in the P group, further validation with larger sample sizes still is needed at a later stage to characterize the lung microbiota of pneumoconiosis complicated with pulmonary infection patients.

More and more studies have found the relationship between viruses and human diseases. Viruses may cause serious respiratory diseases, tumors, and neuropsychiatric related diseases in humans (Gaglia and Munger, 2018; Bjornevik et al., 2022; Domingo and Rovira, 2020), where respiratory tract viral infection is one of the most common diseases in the human worldwide (Zhang et al., 2020). We found more virus species in pneumoconiosis patients in this study, suggesting that patients like this may be more susceptible to viral attack, and the viruses detected were mainly *Human gammaherpesvirus 4* and Human gammaherpesvirus-like viruses. Like other herpesviruses, the above viruses are double-stranded linear DNA viruses that exhibit a biphasic lifecycle, which are carried for life after infection, and overproduce when immunity is low or compromised, leading to human infection. Studies have shown that herpesviridae reactivation is associated with worse clinical outcomes, possibly as a direct cause or as a manifestation of the outcome of exacerbation of diseases (Huang and He, 2020). We only detailed the lung viruses in pneumoconiosis patients, and the relationship between viruses and the development, diagnosis and treatment of pneumoconiosis patients remains to be explored in more studies.

Overall, our study analyzed the differences in pulmonary microorganisms between pneumoconiosis with pulmonary infection and non-pneumoconiosis with pulmonary infection patients and screened for differential flora between the two groups, such as *M. colombiense*, *F. nucleatum* and the genus *Capnocytophaga.* These species could be used as potential biomarkers for the diagnosis of patients with pneumoconiosis with pulmonary infection. In addition, *M. colombiense* was also confirmed to be positively correlated with the number of years of work related to pneumoconiosis, tentatively suggesting a correlation between pneumoconiosis and microorganisms. This study contributes to the understanding of the relationship between microorganisms and pneumoconiosis and provides potential biomarkers for the diagnosis of pneumoconiosis with pulmonary infection, as well as basic data for the investigation of the pathogenesis of the disease.

This study still has some shortcomings. First, this is a single-center study and the patients enrolled only represent the lung microbiome of pneumoconiosis patients around that center. In addition, the number of patients in this cross-sectional study is relatively small due to the reduced number of pneumoconiosis patients and the fact that the patients are scattered in different hospitals, so more centers are needed to participate and enroll more patients to study the lung microbiome of pneumoconiosis in depth.

## 5 Conclusion

In this study, mNGS technology was used to fully expose the microbiome characteristics of the lungs of patients who had pneumoconiosis. Among the bacterial microbiota in the lungs of pneumoconiosis patients, *Streptococcus* were mainly detected, with *Streptococcus pneumoniae* as the main organism. Fungi were mainly detected in *Aspergillus* with *Aspergillus flavus* as the main organism, and the most frequently detected virus was *Human gammaherpesvirus 4*. The P and Non-P groups had different species at the species level, namely *M. colombiense* and *F. nucleatum*, with the former mainly detected in pneumoconiosis patients and the latter mainly in non-pneumoconiosis patients. As a result, we uncovered microbiome characteristics and differences between pneumoconiosis and non-pneumoconiosis with pulmonary infection patients, which provides a good basis for better understanding the relationship between pneumoconiosis and microorganisms, as well as discovering potential biomarkers.

## Data availability statement

The data presented in the study are deposited in the SRA (https://www.ncbi.nlm.nih.gov/sra/) repository, accession number PRJNA985087.

## Ethics statement

The studies involving human participants were reviewed and approved by Ethics Committee of West China Fourth Hospital Sichuan University. The patients/participants provided their written informed consent to participate in this study.

## Author contributions

MZ and XY designed the study and drafted the manuscript. LX collected the patients’ samples and clinical information. ZZS performed the mNGS sequencing and analyzed the data. All the authors read and approved the final manuscript.

## References

[B1] Barnes H. Goh N. S. L. Leong T. L. Hoy R. (2019). Silica-associated lung disease: an old-world exposure in modern industries. Respirology 24 (12), 1165–1175. doi: 10.1111/resp.13695 31517432

[B2] Bjornevik K. Cortese M. Healy B. C. Kuhle J. Mina M. J. Leng Y.. (2022). Longitudinal analysis reveals high prevalence of Epstein-Barr virus associated with multiple sclerosis. Science 375 (6578), 296–301. doi: 10.1126/science.abj8222 35025605

[B3] Cao B. Huang Y. She D. Y. Cheng Q. J. Fan H. Tian X. L.. (2018). Diagnosis and treatment of community-acquired pneumonia in adults: 2016 clinical practice guidelines by the Chinese Thoracic Society, Chinese Medical Association. Clin. Respir. J. 12 (4), 1320–1360. doi: 10.1111/crj.12674 28756639 PMC7162259

[B4] Chen Z. Cheng H. Cai Z. Wei Q. Li J. Liang J.. (2021). Identification of microbiome etiology associated with drug resistance in pleural empyema. Front. Cell Infect. Microbiol. 11. doi: 10.3389/fcimb.2021.637018 PMC8008065 33796482

[B5] Chen H. Liang Y. Wang R. Wu Y. Zhang X. Huang H.. (2023). Metagenomic next-generation sequencing for the diagnosis of Pneumocystis jirovecii Pneumonia in critically pediatric patients. Ann. Clin. Microbiol. Antimicrob. 22 (1), 6. doi: 10.1186/s12941-023-00555-5 36647095 PMC9841943

[B6] Chinese Thoracic Society (2023). [Consensus of clinical pathways of metagenomic next-generation sequencing test in diagnosis of lower respiratory tract infections in China]. Zhonghua Jie He He Hu Xi Za Zhi 46 (4), 322–323. doi: 10.3760/cma.j.cn112147-20220701-00553 36787941

[B7] Chiu C. Y. Miller S. A. J. N. R. G. (2019). Clinical metagenomics. Nat. Rev. Genet. 20 (6), 341–355. doi: 10.1038/s41576-019-0113-7 30918369 PMC6858796

[B8] Dahyot S. Lemee L. Pestel-Caron M. (2017). Description and role of bacteriological techniques in the management of lung infections. Rev. Mal Respir. 34 (10), 1098–1113. doi: 10.1016/j.rmr.2016.07.007 28688757 PMC7134997

[B9] Deng W. Jiang Y. Qin J. Chen G. Lv Y. Lei Y.. (2023). Metagenomic next-generation sequencing assists in the diagnosis of mediastinal aspergillus fumigatus abscess in an immunocompetent patient: a case report and literature review. Infect. Drug Resist. 16, 1865–1874. doi: 10.2147/IDR.S399484 37020798 PMC10069495

[B10] Dewi I. M. Janssen N. A. Rosati D. Bruno M. Netea M. G. Brüggemann R. J.. (2021). Invasive pulmonary aspergillosis associated with viral pneumonitis. Curr. Opin. Microbiol. 62, 21–27. doi: 10.1016/j.mib.2021.04.006 34034082

[B11] D’Humières C. Salmona M. Dellière S. Leo S. Rodriguez C. Angebault C.. (2021). The potential role of clinical metagenomics in infectious diseases: therapeutic perspectives. Drugs 81 (13), 1453–1466. doi: 10.1007/s40265-021-01572-4 34328626 PMC8323086

[B12] Diao Z. Han D. Zhang R. Li J. (2021). Metagenomics next-generation sequencing tests take the stage in the diagnosis of lower respiratory tract infections. J. Adv. Res. 38, 201–212. doi: 10.1016/j.jare.2021.09.012 35572406 PMC9091713

[B13] Dickson R. P. Schultz M. J. van der Poll T. Schouten L. R. Falkowski N. R. Luth J. E.. (2020). Lung microbiota predict clinical outcomes in critically ill patients. Am. J. Respir. Crit. Care Med. 201 (5), 555–563. doi: 10.1164/rccm.201907-1487OC 31973575 PMC7047465

[B14] Domingo J. L. Rovira J. (2020). Effects of air pollutants on the transmission and severity of respiratory viral infections. Environ. Res. 187, 109650. doi: 10.1016/j.envres.2020.109650 32416357 PMC7211639

[B15] Druzhinin V. G. Baranova E. D. Matskova L. V. Demenkov P. S. Volobaev V. P. Larionov A. V.. (2022). Sputum microbiota in coal workers diagnosed with pneumoconiosis as revealed by 16S rRNA gene sequencing. Life 12 (6), 830. doi: 10.3390/life12060830 35743861 PMC9224638

[B16] Esparcia O. Navarro F. Quer M. Coll P. (2008). Lymphadenopathy caused by Mycobacterium colombiense. J. Clin. Microbiol. 46 (5), 1885–1887. doi: 10.1128/JCM.01441-07 18305134 PMC2395067

[B17] Gaglia M. M. Munger K. (2018). More than just oncogenes: mechanisms of tumorigenesis by human viruses. Curr. Opin. Virol. 32, 48–59. doi: 10.1016/j.coviro.2018.09.003 30268926 PMC6405337

[B18] Gao W. Chen H. Jiang H. Wang Q. Tang M. Wang H. S. (2014). Disseminated cutaneous infection caused by Mycobacterium colombiense. Acta Derm. Venereol. 94 (6), 727–728. doi: 10.2340/00015555-1828 24573766

[B19] GBD 2017 Disease and Injury Incidence and Prevalence Collaborators. (2018). Global, regional, and national incidence, prevalence, and years lived with disability for 354 diseases and injuries for 195 countries and territories, 1990–2017: a systematic analysis for the Global Burden of Disease Study 2017. Lancet (London, England), 392 (10159), 1789–1858. doi: 10.1016/S0140-6736(18)32279-7 PMC6227754 30496104

[B20] GBD 2013 Mortality and Causes of Death Collaborators. (2015). Global, regional, and national age-sex specific all-cause and cause-specific mortality for 240 causes of death, 1990–2013: a systematic analysis for the Global Burden of Disease Study 2013. Lancet 385 (9963), 117–117. doi: 10.1016/S0140-6736(14)61682-2 25530442 PMC4340604

[B21] GraphPad Software Inc (2019). GraphPad prism version 8.0.2 for windows (San Diego, CA: GraphPad Software Inc).

[B22] Guo Y. L. Chen Y. Q. Wang K. Qin S. M. Wu C. Kong J. L. (2010). Accuracy of BAL galactomannan in diagnosing invasive aspergillosis: a bivariate metaanalysis and systematic review. Chest 138 (4), 817–824. doi: 10.1378/chest.10-0488 20453070

[B23] Hammond E. E. McDonald C. S. Vestbo J. Denning D. W. (2020). The global impact of Aspergillus infection on COPD. BMC Pulm. Med. 20 (1), 241. doi: 10.1186/s12890-020-01259-8 32912168 PMC7488557

[B24] Honma K. Abraham J. L. Chiyotani K. De Vuyst P. Dumortier P. Gibbs A. R.. (2004). Proposed criteria for mixed-dust pneumoconiosis: definition, descriptions, and guidelines for pathologic diagnosis and clinical correlation. Hum. Pathol. 35 (12), 1515–1523. doi: 10.1016/j.humpath.2004.09.008 15619211

[B25] Huang H. He H. (2020). Herpesviridae reactivation for poor outcome in ARDS patients with ECMO: criminal or witness? Ann. Intensive Care 10 (1), 10. doi: 10.1186/s13613-020-0626-4 31993803 PMC6987285

[B26] IBM Corp. (2017). IBM SPSS Statistics for windows, version 25.0 (Armonk, NY: IBM Corp).

[B27] Ju C. R. Lian Q. Y. Guan W. J. Chen A. Zhang J. H. Xu X.. (2022). Metagenomic next-generation sequencing for diagnosing infections in lung transplant recipients: a retrospective study. Transpl. Int. 35. doi: 10.3389/ti.2022.10265 PMC8866178 35221789

[B28] Jun J. S. Jung J. I. Kim H. R. Ahn M. I. Han D. H. Ko J. M.. (2013). Complications of pneumoconiosis: radiologic overview. Eur. J. Radiol. 82 (10), 1819–1830. doi: 10.1016/j.ejrad.2013.05.026 23791520

[B29] Kim Y. M. Kim M. Kim S. K. Park K. Jin S. H. Lee U. S.. (2009). Mycobacterial infections in coal workers' pneumoconiosis patients in South Korea. Scand. J. Infect. Dis. 41 (9), 656–662. doi: 10.1080/00365540903089468 19565408

[B30] Kruskal W. H. Wallis W. A. (1952). Use of ranks in one-criterion variance analysis. J. Am. Stat. Assoc. 47 (260), 583–621. doi: 10.1080/01621459.1952.10483441

[B31] Langelier C. Kalantar K. L. Moazed F. Wilson M. R. Crawford E. D. Deiss T.. (2018). Integrating host response and unbiased microbe detection for lower respiratory tract infection diagnosis in critically ill adults. Proc. Natl. Acad. Sci. USA 115 (52), E12353–E12362. doi: 10.1073/pnas.1809700115 30482864 PMC6310811

[B32] Langmead B. Salzberg S. L. (2012). Fast gapped-read alignment with Bowtie 2. Nat. Methods 9 (4), 357–359. doi: 10.1038/nmeth.1923 22388286 PMC3322381

[B33] Larry M. R. Daniel M. R. Santiago O. N. (2019). Ganglionar Mycobacteriosis associated to Mycobacterium colombiense in a Seropositive HIV Patient. Case report a literature review. J. AIDS & Clinical Research. 10 (4). 1000792.

[B34] Levy L. Juvet S. C. Boonstra K. Singer L. G. Azad S. Joe B.. (2018). Sequential broncho-alveolar lavages reflect distinct pulmonary compartments: clinical and research implications in lung transplantation. Respir. Res. 19 (1), 102. doi: 10.1186/s12931-018-0786-z 29801490 PMC5970521

[B35] Li W. J. Guo Y. L. Liu T. J. Wang K. Kong J. L. (2015). Diagnosis of pneumocystis pneumonia using serum (1-3)-β-D-Glucan: a bivariate meta-analysis and systematic review. J. Thorac. Dis. 7 (12), 2214–2225. doi: 10.3978/j.issn.2072-1439.2015.12.27 26793343 PMC4703639

[B36] Li Y. Xiao K. Xiao S. Wang M. Pei S. Liu H.. (2022). Difference in intestinal flora and characteristics of plasma metabonomics in pneumoconiosis patients. Metabolites 12 (10), 917. doi: 10.3390/metabo12100917 36295819 PMC9609413

[B37] Li J. Zhou L. Gong X. Wang Y. Yao D. Li H. (2022). Abiotrophia defectiva as a rare cause of mitral valve infective endocarditis with mesenteric arterial branch pseudoaneurysm, splenic infarction, and renal infarction: a case report. Front. Med. (Lausanne) 9. doi: 10.3389/fmed.2022.780828 PMC8962948 35360716

[B38] Liu W. Liang R. Zhang R. Wang B. Cao S. Wang X.. (2022). Prevalence of coal worker's pneumoconiosis: a systematic review and meta-analysis. Environ. Sci. pollut. Res. Int. 29 (59), 88690–88698. doi: 10.1007/s11356-022-21966-5 35836046

[B39] Lu Y. Chen Y. Q. Guo Y. L. Qin S. M. Wu C. Wang K. (2011). Diagnosis of invasive fungal disease using serum (1→3)-β-D-glucan: a bivariate meta-analysis. Intern. Med. 50 (22), 2783–2791. doi: 10.2169/internalmedicine.50.6175 22082890

[B40] Mac Aogáin M. Narayana J. K. Tiew P. Y. Ali N. A. B. M. Yong V. F. L. Jaggi T. K.. (2021). Integrative microbiomics in bronchiectasis exacerbations. Nat. Med. 27 (4), 688–699. doi: 10.1038/s41591-021-01289-7 33820995

[B41] McGrath E. E. Bardsley P. (2009). An association between Mycobacterium malmoense and coal workers' pneumoconiosis. Lung 187 (1), 51–54. doi: 10.1007/s00408-008-9104-8 18758857

[B42] Miao Q. Ma Y. Wang Q. Pan J. Zhang Y. Jin W.. (2018). Microbiological diagnostic performance of metagenomic next-generation sequencing when applied to clinical practice. Clin. Infect. Dis. 67 (suppl_2), S231–S240. doi: 10.1093/cid/ciy693 30423048

[B43] Miller S. Naccache S. N. Samayoa E. Messacar K. Arevalo S. Federman S.. (2019). Laboratory validation of a clinical metagenomic sequencing assay for pathogen detection in cerebrospinal fluid. Genome Res. 29 (5), 831–842. doi: 10.1101/gr.238170.118 30992304 PMC6499319

[B44] Mingjing Chen T. L. Li S. Zuo H. Pei X. (2017). Drug resistance of pathogens and epidemiological analysis on 348 cases of silicosis with pulmonary infections. Modern Prev. Med. 44 (13), 5.

[B45] Murcia M. I. Tortoli E. Menendez M. C. Palenque E. Garcia M. J. (2006). Mycobacterium colombiense sp. nov., a novel member of the Mycobacterium avium complex and description of MAC-X as a new ITS genetic variant. Int. J. Syst. Evol. Microbiol. 56 (Pt 9), 2049–2054. doi: 10.1099/ijs.0.64190-0 16957098

[B46] Pena E. Machado D. Viveiros M. Jordão S. (2019). A case report of disseminated Mycobacterium colombiense infection in an HIV patient. Int. J. Mycobacteriol. 8 (3), 295–297. doi: 10.4103/ijmy.ijmy_100_19 31512608

[B47] Perret J. L. Plush B. Lachapelle P. Hinks T. S. Walter C. Clarke P.. (2017). Coal mine dust lung disease in the modern era. Respirology (Carlton, Vic.) 22 (4), 662–670. doi: 10.1111/resp.13034 28370783

[B48] Poulin S. Corbeil C. Nguyen M. St-Denis A. Côté L. Le Deist F.. (2013). Fatal Mycobacterium colombiense/cytomegalovirus coinfection associated with acquired immunodeficiency due to autoantibodies against interferon gamma: a case report. BMC Infect. Dis. 13, 24. doi: 10.1186/1471-2334-13-24 23336346 PMC3561114

[B49] Qi X. M. Luo Y. Song M. Y. Liu Y. Shu T. Liu Y.. (2021). Pneumoconiosis: current status and future prospects. Chin. Med. J. (Engl.) 134 (8), 898–907. doi: 10.1097/CM9.0000000000001461 33879753 PMC8078400

[B50] Qian L. Shi Y. Li F. Wang Y. Ma M. Zhang Y.. (2020). Metagenomic next-generation sequencing of cerebrospinal fluid for the diagnosis of external ventricular and lumbar drainage-associated ventriculitis and meningitis. Front. Microbiol. 11. doi: 10.3389/fmicb.2020.596175 PMC7767851 33381092

[B51] Ramachandran P. S. Ramesh A. Creswell F. V. Wapniarski A. Narendra R. Quinn C. M.. (2022). Integrating central nervous system metagenomics and host response for diagnosis of tuberculosis meningitis and its mimics. Nat. Commun. 13 (1), 1675. doi: 10.1038/s41467-022-29353-x 35354815 PMC8967864

[B52] R Core Team (2021). R: a language and environment for statistical computing (Vienna, Austria: R Foundation for Statistical Computing).

[B53] Rudramurthy S. M. Paul R. A. Chakrabarti A. Mouton J. W. Meis J. F. (2019). Invasive aspergillosis by aspergillus flavus: epidemiology, diagnosis, antifungal resistance, and management. J. Fungi (Basel) 5 (3), 55. doi: 10.3390/jof5030055 31266196 PMC6787648

[B54] Shi Y. Huang Y. Zhang T. T. Cao B. Wang H. Zhuo C.. (2019). Chinese Guidelines for the diagnosis and treatment of hospital-acquired pneumonia and ventilator-associated pneumonia in adults, (2018 Edition). J. Thorac. Dis. 11 (6), 2581–2616. doi: 10.21037/jtd.2019.06.09 31372297 PMC6626807

[B55] Shi P. Xing X. Xi S. Jing H. Yuan J. Fu Z.. (2020). Trends in global, regional and national incidence of pneumoconiosis caused by different aetiologies: an analysis from the Global Burden of Disease Study 2017. Occup. Environ. Med. 77 (6), 407–414. doi: 10.1136/oemed-2019-106321 32188634

[B56] Tang M. Zeng W. Qiu Y. Fang G. Pan M. Li W.. (2023). Clinical features of rare disseminated mycobacterium colombiense infection in nine patients who are HIV-negative in Guangxi, China. Int. J. Infect. Dis. 128, 321–324. doi: 10.1016/j.ijid.2023.01.002 36642210

[B57] Vandewoude K. Blot S. Benoit D. Depuydt P. Vogelaers D. Colardyn F. (2004). Invasive aspergillosis in critically ill patients: analysis of risk factors for acquisition and mortality. Acta Clin. Belg. 59 (5), 251–257. doi: 10.1179/acb.2004.037 15641394

[B58] Vangara A. Gudipati M. Chan R. Do T. V. Bawa O. Shyam Ganti S.. (2022). Chronic pulmonary aspergillosis infection in coal workers pneumoconiosis with progressive massive fibrosis. J. Investig. Med. High Impact Case Rep. 10, 23247096221127100. doi: 10.1177/23247096221127100 PMC9516416 36154322

[B59] Van Ingen J. Turenne C. Y. Tortoli E. Wallace R. J. Jr Brown-Elliott B. A. (2018). A definition of the Mycobacterium avium complex for taxonomical and clinical purposes, a review. Int. J. Syst. Evol. Microbiol. 68 (11), 3666–3677. doi: 10.1099/ijsem.0.003026 30231956

[B60] Vuorenmaa K. Ben Salah I. Barlogis V. Chambost H. Drancourt M. (2009). Mycobacterium colombiense and pseudotuberculous lymphadenopathy. Emerg. Infect. Dis. 15 (4), 619–620. doi: 10.3201/eid1504.081436 19331753 PMC2671429

[B61] Wang T. Hou Y. Wang R. (2019). A case report of community-acquired pseudomonas aeruginosa pneumonia complicated with MODS in a previously healthy patient and related literature review. BMC Infect. Dis. 19 (1), 1–6. doi: 10.1186/s12879-019-3765-1 30736735 PMC6368713

[B62] Wood D. E. Lu J. Langmead B. (2019). Improved metagenomic analysis with Kraken 2. Genome Biol. 20 (1), 257. doi: 10.1186/s13059-019-1891-0 31779668 PMC6883579

[B63] Wu L. Zeng T. Deligios M. Milanesi L. Langille M. G. I. Zinellu A.. (2020). Age-related variation of bacterial and fungal communities in different body habitats across the young, elderly, and centenarians in Sardinia. Msphere 5 (1), e00558–19. doi: 10.1128/mSphere.00558-19 PMC7045387 32102941

[B64] Xin Y. Zhang N. (2017). [The analysis of pathogens distribution and drug resistance of bacteria in sputum samples of pneumoconiosis patients combined with lower respiratory tract infection.]. Zhonghua Lao Dong Wei Sheng Zhi Ye Bing Za Zhi 35 (1), 58–61. doi: 10.3760/cma.j.issn.1001-9391.2017.01.015 28241707

[B65] Xu J. Zhou P. Liu J. Zhao L. Fu H. Han Q.. (2023). Utilizing metagenomic next-generation sequencing (mNGS) for rapid pathogen identification and to inform clinical decision-making: results from a Large real-world cohort. Infect. Dis. Ther. 12 (4), 1175–1187. doi: 10.1007/s40121-023-00790-5 36988865 PMC10147866

[B66] Yu X. Jiang W. (2021). Mycobacterium colombiense and Mycobacterium avium complex causing severe pneumonia in a patient with HIV identified by a novel molecular-based method. Infect. Drug Resist. 14, 11–16. doi: 10.2147/IDR.S282190 33442272 PMC7797356

[B67] Zeng X. Wu J. Li X. Xiong W. Tang L. Li X.. (2022). Application of metagenomic next-generation sequencing in the etiological diagnosis of infective endocarditis during the perioperative period of cardiac surgery: a prospective cohort study. Front. Cardiovasc. Med. 9. doi: 10.3389/fcvm.2022.811492 PMC8965566 35369282

[B68] Zhang N. Wang L. Deng X. Liang R. Su M. He C.. (2020). Recent advances in the detection of respiratory virus infection in humans. J. Med. Virol. 92 (4), 408–417. doi: 10.1002/jmv.25674 31944312 PMC7166954

[B69] Zhimin Ma W. B. (2020). Distribution of pathogens and their drug resistance in occupational pneumoconiosis with pulmonary infections. Med. Diet Health 18 (7), 2.

